# Pharmacologic Ascorbate in Myeloma Treatment: Doses Matter

**DOI:** 10.1016/j.ebiom.2017.03.014

**Published:** 2017-03-09

**Authors:** Pierre-Christian Violet, Mark Levine

**Affiliations:** Molecular and Clinical Nutrition Section, NIDDK, NIH, Bethesda, MD 20892, United States

Ascorbic acid (vitamin C) has followed opaque paths in treating multiple myeloma, and other malignancies. In myeloma cells, ascorbate surprisingly depleted glutathione and potentiated arsenate trioxide efficacy. Because oral ascorbate at 1 g produces plasma saturation, this dose was selected with arsenate. Predictably, in small trials there was neither benefit nor harm (Rollig and Illmer, 2009, Parrow et al., 2013). Concurrently, the emerging proteasome inhibitor bortezomib was inhibited by ascorbate, which complexed with bortezomib's boron group. Despite inconsistencies (Perrone et al., 2009, Bannerman et al., 2011), concern remains about ascorbate-bortezomib interaction, and bortezomib is one of few agents that perhaps is ascorbate-inhibitable in vivo.

Independently, pharmacologic ascorbate has become a promising chemotherapeutic agent in cancer treatment, but again following an opaque course (Parrow et al., 2013). Proposed as an anticancer treatment in 1954, and despite encouraging case documentation, ascorbate had no efficacy in two double-blind placebo-controlled trials. Ascorbate pharmacokinetics, from clinical studies on healthy subjects, explained why. Tight-control of ascorbate concentrations from oral ingestion, as in the double-blind trials, is by-passed by intravenous administration, as in the documented cases (Padayatty et al., 2004, Parrow et al., 2013). Only intravenous ascorbate at pharmacologic doses, (i.e., 1 g/kg) produces up to 25 mM plasma and extracellular fluid concentrations, the latter serving as a pro-drug for extracellular H_2_O_2_ formation (Chen et al., 2008). With H_2_O_2_ + millimolar ascorbate, reactive oxygen species formed by Fenton-type reactions kill cancer but not normal cells. With proper patient screening, pharmacologic ascorbate has minimal risk (Padayatty et al., 2010). Many reports document efficacy of pharmacologic ascorbate in pre-clinical solid-tumor models and in phase I/early phase II trials.

In this issue of *EBioMedicine*, Xia and colleagues use pharmacologic ascorbate in pre-clinical studies of myeloma (Xia et al., 2017). Advances presented included investigation of myeloma and non-myeloma human bone marrow cells from patients with myeloma; with smoldering myeloma; and with monoclonal gammopathy of uncertain significance. Iron homeostasis was characterized in bone marrow cells from several hundred patients, and findings were consistent with effects of pharmacologic ascorbate. Because myeloma cells are iron-rich, increasing iron export increased their resistance to pharmacologic ascorbate. Animal model results showed that pharmacologic ascorbate was synergistic with melphalan, permitting a 5-fold dose reduction. Consistent with mechanistic understanding, extracellular H_2_O_2_ generated by pharmacologic ascorbate was necessary for efficacy (Chen et al., 2008, Parrow et al., 2013). Doses mattered (Chen et al., 2008, Parrow et al., 2013): compared to myeloma studies above, Xia and colleagues used doses several hundred-fold higher. Xia and colleagues recognized that higher intraperitoneal doses compared to intravenous doses were required to achieve equivalent pharmacologic concentrations (Chen et al., 2008, Parrow et al., 2013).

Although median overall survival for multiple myeloma has improved to about 5 years, there is constant effort to increase survival (Durie et al., 2017). Myeloma therapies are based on co-morbidities, age, staging, prior treatments, and individualized risk assessment. Given continued use of melphalan in myeloma management, melphalan complications, and the compelling melphalan-sparing described by Xia et al. (Xia et al., 2017), it is worth advancing pharmacologic ascorbate + melphalan to phase I trials. Pharmacologic ascorbate has additive or synergistic promise with other therapies in myeloma, even bortezomib, if dosage and timing with other agents are considered with emerging knowledge and support from preclinical models. Other iron-rich tumor cells, such as lymphomas, are ripe for pharmacologic ascorbate testing. The promise of pharmacologic ascorbate is that it provides multiple effector mechanisms for cancer cell death once extracellular H_2_O_2_ is generated (Parrow et al., 2013), even if resistance develops to one pathway.

Although pharmacologic ascorbate has low risk, renal insufficiency is one contraindication (Parrow et al., 2013). It is uncertain whether pharmacologic ascorbate will precipitate renal insufficiency in myeloma patients with proteinuria, and this can be addressed in phase I clinical studies. Phase I trial design should consider intended duration of therapy: whether intent of pharmacologic ascorbate use is short-term (weeks), i.e., in preparation for autologous stem cell transplant, vs. months for maintenance therapy. Downsides of pharmacologic ascorbate are minimal compared to other treatments if patients are screened for normal renal function, iron overload, glucose-6-phosphate dehydrogenase deficiency, and prior oxalate nephropathy. The findings from Xia et al. exemplify how data from preclinical studies can guide the way for future clinical trials of pharmacological ascorbate in multiple myeloma and other bone-marrow neoplasms.

## Disclosure

No conflicts of interest to declare.
